# Impact of the COVID-19 Pandemic on Children With Neurodevelopmental Disorders When School Closures Were Lifted

**DOI:** 10.3389/fped.2021.789045

**Published:** 2021-12-13

**Authors:** Kota Suzuki, Michio Hiratani

**Affiliations:** ^1^Faculty of Education, Shitennoji University, Habikino, Japan; ^2^Hiratani Child Development Clinic, Fukui, Japan

**Keywords:** coronavirus disease 2019, neurodevelopmental disorder, behavioral problem, school closure, longitudinal data

## Abstract

Human activities have been changing in conjunction with the status of the coronavirus disease 2019 (COVID-19) pandemic, with school closures and activity cancellations becoming commonplace. As such, the COVID-19 pandemic likely also has had a detrimental impact on the behavioral problems of children with neurodevelopmental disorders (NDD). In Japan, the government issued a stay-at-home order causing children to stop participating in school activities following the first declaration of a state of emergency (April 7 to May 25, 2020). During winter 2020, the stay-at-home order and school closures were lifted. Using longitudinal data of children with NDD, we compared the behavioral problems of children with NDD between October 1 and December 31, 2020 (i.e., winter 2020) with their behavioral problems before the COVID-19 pandemic (pre-COVID-19). In this study, 143 caregivers of children with NDD evaluated their behavioral problems using the Japanese version of the Aberrant Behavior Checklist (ABC-J) in the pre-COVID-19 period and winter 2020. We found no scores that were higher in winter 2020 compared to pre-COVID-19. Moreover, irritability and hyperactivity scores were significantly lower in winter 2020 compared to pre-COVID-19. These findings suggest weak negative impacts of the COVID-19 pandemic on the behavioral problems of children with NDD. In schools and clinical practice, children learned knowledge about COVID-19 and related preventive behaviors. Therefore, these practices may have alleviated the negative impact of the COVID-19 pandemic on children with NDD.

## Introduction

The coronavirus disease 2019 (COVID-19) pandemic has undoubtedly changed human activities on a global scale. In Japan, a state of emergency was declared from April 7 to May 25, 2020. During that period, the government issued a stay-at-home order, and children stopped participating in school activities. After the state of emergency was lifted, children resumed their school activities. However, the government recommended preventive behavior practices, and children in schools had to put on a mask and social distance from each other.

The COVID-19 pandemic and the subsequent changes induced in human activities have been influencing individuals' mental health (1). For example, in a longitudinal survey performed in the UK, the level of reported individuals' mental health was found to be inferior during the period of COVID-19 lockdown (April, 2020) compared to before the COVID-19 pandemic (2). Additionally, Chilean caregivers reported that children were more affectionate, more restless, and more frustrated during the COVID-19 pandemic than before the pandemic (3). In contrast, a positive effect was reported in a previous American-based study, where the score of perceived social support was increased by stay-at-home or shelter-in-place orders (4). The monthly suicide rate in Japan was decreased in the first 5 months of the COVID-19 pandemic (February to June 2020) compared to the same period in previous years (February to June 2019); however, the rate increased between July and October 2020 (5), suggesting that the impact of COVID-19 varies with time.

Children with neurodevelopmental disorders (NDD) such as attention deficit hyperactivity disorder (ADHD), autism spectrum disorder (ASD), and intellectual disability (ID), are at a higher risk of developing mental health and behavioral problems (6, 7). Hence, it is possible that children with NDD are more susceptible to the effects of COVID-19 pandemic, as well as to the subsequent changes in human activities, in comparison to neurotypical children. A previously performed Japanese longitudinal study showed that scores of externalizing problems and aggressive behavior in children with NDD were higher during the stay-at-home order and school closures (May 2020) compared to before the COVID-19 pandemic (8). In an American longitudinal study where half of the participants were diagnosed with ADHD, scores of depression, anxiety, and oppositionality/defiance increased during the stay-at-home order compared to before the COVID-19 pandemic; however, these scores were subsequently reversed when the stay-at-home order was lifted (9). Although previous studies have underlined the impact of the COVID-19 pandemic on mental health and behavioral problems in children with NDD (8, 9), longitudinal findings focusing on children with NDD are not sufficient. Moreover, the status of the COVID-19 infection and subsequent government responses varies among countries, regions, and time. Hence, it was important for the future pandemic to examine the impacts of COVID-19 pandemic in several regions. In this study, we report longitudinal findings in Fukui Prefecture due to the impact of the COVID-19 pandemic on behavioral and mental health problems in children with NDD once the stay-at-home order and school closures were lifted. Fukui Prefecture is a provincial area of Japan. Although the case numbers of COVID-19 were small, a stay-at-home order was issued according to the first declaration of a state of emergency (April 7 to May 25, 2020) by the Japanese government. After this period, children resumed their school activities in Fukui Prefecture, and school closures and stay-at-home orders were no longer issued. We considered that the situations regarding the COVID-19 pandemic were unique for each region of the previous studies (8, 9).

## Methods

### Participants

Children with NDD and their caregivers were recruited from a developmental clinic in Fukui Prefecture for a survey of the effects of COVID-19 on behavioral and mental health problems of children with NDD. About 4,000 children with NDD have used the clinic, and over 50 new children used the clinic due to exacerbated NDD symptoms every month. The inclusion criteria of the survey were the following; (1) caregivers and children who visited the NDD section of the clinic between October 1 and December 31, 2020 (i.e., winter 2020); (2) the ages of the children ranged from 6 to 18 years. A portion of the participants' data was also used in a study by Suzuki and Hiratani (10). The Japanese version of the Aberrant Behavior Checklist (ABC-J) (11) captures behavioral problems related to the wide variety of NDDs, e.g., ID, ADHD, and ASD. Within the clinic, ABC-J is included in the first assessment, and is frequently used for the evaluation of the need for medication and other interventions. In this study, children with a wide variety of NDDs participated, and we could not prospectively investigate the effects of COVID-19 on behavior; thus, we used ABC-J for evaluating behavioral problems instead. In addition, we set the pre-COVID-19 period as 2 years between April 2018 and April 2020 because a shorter period is easier to manage and not associated with any major developmental changes. The period of winter 2020 was set as a three month period because of the situation of the COVID-19 pandemic and the recruitment period for the participants.

In this study, we used questionnaire data gathered from responses by 294 caregivers. One hundred and eighty-two caregivers were evaluated after excluding incomplete responses and data from 143 caregivers was used for the analysis. If ABC-Js were evaluated multiple times during the pre-COVID-19 range, we used the last, most recent scores. All caregivers provided written informed consent before participation. We explained the overview of this study for children with the easy-to-understand explanation form. Children also provided written informed consent (*n* = 97) or informed assent (*n* = 157), if possible. The study design was approved by the ethics committee of Shitennoji University (approval number: 2020-17).

### Japanese Version of the Aberrant Behavior Checklist (ABC-J)

Caregivers rated the behavioral problems of their children using ABC-J criteria. ABC was originally developed for assessing the treatment effects for individuals with severe ID (12), and has also been used for evaluating behavioral problems in children with NDD (13). ABC-J consists of 58 items which are classified as the following five subscales (11, 12): “irritability,” “lethargy,” “stereotypic behavior,” “hyperactivity,” and “inappropriate speech.” Caregivers rated children's behavior using a four-point scale (no problem = 0, remarkable problem = 4). In our study, participants' data were excluded from the analysis when there was more than one missing value in each subscale. In contrast, when there was only one missing value in a subscale (e.g., item 1: 1, item 2: missing, item 3: 2, item 4: 2, item 5: 1). We calculated the mean values for each item of the subscale, excluding the missing values, and then used the mean value as the score for the item with a missing value (e.g., item 1: 1, item 2: *1.5* (missing), item 3: 2, item 4: 2, item 5: 1), with reference to the other scales, e.g., (14). Finally, we summed the score of all the subscales. In data of 14 of the participants, the mean value was used as the score for the item with a missing value.

### Statistical Analysis

The differences in scores between pre-COVID-19 and winter 2020 periods were evaluated by paired *t*-tests. *P*-values were corrected by the Bonferroni method. Effect sizes were calculated as Hedges' *g*. Additionally, we performed analyses of variance (ANOVAs) on scores with diagnosis of ADHD, diagnosis of ASD, and COVID-19 pandemic (winter 2020 and pre-COVID-19) to test the differences of effects among diagnoses. We did not use the other diagnoses as independent variables because of small numbers. Moreover, the Pearson's correlational tests were performed between difference score (winter 2020—pre-COVID-19) and IQ, to test the relationship between the impact of COVID-19 pandemic and IQ. Statistical analysis was performed using R version 3.52 (15).

## Results

Characteristics of participants are shown in Tables 1, 2. Most children were diagnosed with ADHD and/or ASD. There were more boys than girls present in this study. The ages of the children ranged from 6 to 16 years [mean ± standard deviation (SD) = 9.76 ± 2.36 years], and their IQs ranged from 18 to 138 (mean ± SD = 89.02 ± 19.20).

**Table 1:** Diagnosis of children (*n*).

	**Comorbidity**		
	**No**	**SLD**	**ID**
ADHD + ASD	24	32	1
ADHD	17	31	2
ASD	9	6	9
SLD	3	NA	NA
ID	4	NA	NA
Other	5	NA	NA

*ADHD, attention-deficit/hyperactivity disorder; ASD, autism spectrum disorder; SLD, specific learning disorder; ID, intellectual disability; NA, not available*.

**Table 2:** Characteristics of participants.

Age of children (years)	M	9.76
	SD	2.36
IQ	M	89.02
	SD	19.20
Gender (*n*)	Boys	118
	Girls	25
Age of caregivers (years)	M	40.29
	SD	5.63
Respondent (*n*)	Mother	126
	Father	17

*IQ, intelligence quotient; M, mean; SD, standard deviation*.

Table 3 shows the means of the scores for each ABC-J subscale in the pre-COVID-19 period and in winter 2020. There were no subscales for which the mean value was higher in winter 2020 compared to pre-COVID-19. Furthermore, the irritability score was significantly lower in winter 2020 as compared to pre-COVID-19 [*t*_(142)_ = 3.66, Bonferroni corrected *p* = 0.001, Hedges' *g* = 0.21]. The hyperactivity score was also significantly lower in winter 2020 than pre-COVID-19 [*t*_(142)_ = 4.31, Bonferroni corrected *p* < 0.001, Hedges' *g* = 0.25]. However, there were no significant differences between the pre-COVID-19 and winter 2020 scores for lethargy [*t*_(142)_ = 0.94, uncorrected *p* = 0.35, Hedges' *g* = 0.06], stereotypic behavior [*t*_(142)_ = 0.97, uncorrected *p* = 0.33, Hedges' *g* = 0.06], and inappropriate speech [*t*_(142)_ = 0.96, uncorrected *p* = 0.34, Hedges' *g* = 0.05]. There were not any interactions between COVID-19 pandemic and the diagnoses of ADHD and ASD on scores (all *ps* > 0.05). There were no significant correlations between difference scores and IQ (*rs* = −0.11 to 0.05, all *ps* > 0.13).

**Table 3:** The means of the scores for each Aberrant Behavior Checklist (ABC-J) subscale in pre-COVID-19 period (April 2018 to February 2020) and winter 2020 (October to December 2020).

		**Pre-COVID-19**	**Winter 2020**	***p***
Irritability	M	8.52	6.37	^**^
	SD	7.90	6.55	
Lethargy	M	4.82	4.36	
	SD	5.94	5.85	
Stereotypic behavior	M	1.98	1.73	
	SD	3.12	2.65	
Hyperactivity	M	11.59	8.57	^***^
	SD	9.43	7.50	
Inappropriate speech	M	1.72	1.56	
	SD	2.42	2.10	

* *p < 0.05*,

** *p < 0.01*,

*** *p < 0.001 (Bonferroni corrected), M, Mean; SD, standard deviation*.

## Discussion

In this study, we found that none of the children participating in this study scored higher on the five subscales during the winter 2020 period than the pre-COVID-19 period. In a previous study on children with and without ADHD (9), behavioral, and mental health problems increased from the pre-COVID-19 period to when the stay-at-home order was issued; however, this increase was not sustained after the order was lifted. In the Fukui Prefecture, citizens were not subjected to a stay-at-home order during winter 2020, and children continued to participate in school activities. Moreover, there was only a small number of COVID-19 infection cases (111 people) in the Fukui Prefecture (16). Therefore, we consider the negative impact of the COVID-19 pandemic on the behavioral and mental health problems of children with NDD to be weak.

The present results indicate a distinct improvement in irritability and hyperactivity after the COVID-19 pandemic, and we identified three factors that appear to be associated with this improvement. Firstly, we considered the stay-at-home order and school closure as a factor. In the first declared state of emergency, most families spent time together, which potentially led to the development of more intimate relationships between children and caregivers. Secondly, the long period of social distancing might have been a contributing factor to this improvement as it may have limited any potential troublesome relationships between children with NDD and their classmates. Thirdly, measures to prevent infection at school might have had a positive impact on some children with NDD. School activities were structured to prevent infection, and this situation might have made life more comfortable for children with NDD.

The results seen here may reflect the effect of clinical intervention. Since we used scores of the last evaluation of the ABC-J in the pre-COVID-19 period, the effects of medication did not influence the present results; however, it was possible that several interventions of clinicians (e.g., advice, counseling, supportive relationships) improved behavioral problems beforehand. A previous study of children in a special education class indicated that the scores of ABC were not different between 6 and10 years children and 10–14 years children (17). Although our findings are speculated to not be related to the effects of intervention, the effects of the COVID-19 pandemic were not completely dissociated from the effects of the intervention.

Participants were recruited from a single clinic; thus, our results were influenced by the situation of the clinic. The case number was much smaller in Fukui Prefecture than in metropolitan areas of Japan and other countries (16); however, media conveyed information about the risks of COVID-19 and the necessary of preventive behavior daily. These situations were not identical to other areas. A previous study showed that scores of depression, anxiety, and oppositionality/defiance increased during the stay-at-home order compared to before the COVID-19 pandemic, whereas these scores were subsequently reduced when the stay-at-home order was lifted (9). Therefore, the weak negative impact of the COVID-19 pandemic might be a common occurrence when the stay-at-home order and school closures were lifted.

Children with wide variety of NDDs (e.g., ADHD and ASD) were participated, and the range of IQ was also wide (IQ: 18–138). There were not any significant interactions between COVID-19 pandemic and diagnoses, and significant correlations between difference score (winter 2020—pre-COVID-19) and IQ. These findings suggested that the variety of NDDs and IQ did not influence the main results. However, it was possible that the factors contributing to the results were different among diagnoses and the range of IQ. For example, children with ASD might prefer the structured school activity, whereas children with severe ID might not understand the situation of COVID-19 well.

In this study, there were fewer girls than boys. The result was consistent with the finding that the proportion of boys is larger in ASD and ADHD (18). (19) reported that women experienced significantly greater fear of COVID-19 than men. Thus, the larger proportion of boys might be associated with the weak impact of COVID-19. It was also possible that the caregivers were influenced by the COVID-19 pandemic, which confounded evaluation using the ABC-J. This problem may be partly resolved by using the data of teacher's rating, but there was insufficient data of teacher ratings in the clinic. We expected that other clinics and institutions will likely report the findings based on teacher rating data. In this study, the survey was not performed during the stay-at-home order and school closure. Thus, it was not clear whether the impacts of COVID-19 pandemic were weak in Fukui Prefecture, or children with NDD were recovered during winter 2020.

Our previous study (10) indicated that children's depressive symptoms were associated with caregivers' worry about the relationship between the children's activity and COVID-19 infection. Unsurprisingly, parenting stress was increased by school closures due to the initial declaration of a state of emergency (20). Another previous study suggested a potential increase in the risk of maltreatment due to the added stress of the COVID-19 pandemic (21). Tellingly, the monthly suicide rate did increase after the end of the first state of emergency (5). These findings, taken together, suggest that there is a definite negative impact of the COVID-19 pandemic on behavioral problems in general, with parents and caregivers undergoing a singularly unique set of stressors. The impact of the COVID-19 pandemic was not as strong on children's behavioral states as expected, possibly due to informational material being spread around the community in a prompt response to the pandemic. An educational guide was developed to teach children about COVID-19 and the related preventive behaviors needed for school (22). Kawabe et al. (23) developed brochures specifically to educate children with ASD on preventive behavior related to the spread of COVID-19. Therefore, these practices may have alleviated the negative impact the COVID-19 pandemic had on children's behavior. We expect that clinicians will report their practices, and the relationship between our findings and the practices will be clarified, providing important data for use in future pandemics.

## Data Availability Statement

The datasets presented in this article are not readily available because no informed consent was given by the participants for open data sharing. The data that support the findings of this study are available on request from Kota Suzuki, kt.suzuki@hotmail.co.jp, and require approval by the Ethics Committee of Shitennoji University.

## Ethics Statement

The studies involving human participants were reviewed and approved by the Ethics Committee of the Shitennoji University (approval number: 2020-17). All caregivers provided written informed consent before participation. Children also provided written informed consent or informed assent, if possible.

## Author Contributions

KS contributed to the study design, interpretation of data, and writing the manuscript. MH contributed to the study design, data collection, interpretation of data, and writing the manuscript. Both authors contributed to the article and approved the submitted version.

## Funding

This study was partly supported by a group research grant from Shitennoji University and a Grant-in-Aid (KAKENHI) for Scientific Research (C) (grant number 19K03304) from Japan Society for the Promotion of Science (JSPS).

## Conflict of Interest

The authors declare that the research was conducted in the absence of any commercial or financial relationships that could be construed as a potential conflict of interest.

## Publisher's Note

All claims expressed in this article are solely those of the authors and do not necessarily represent those of their affiliated organizations, or those of the publisher, the editors and the reviewers. Any product that may be evaluated in this article, or claim that may be made by its manufacturer, is not guaranteed or endorsed by the publisher.
